# Breast density measurements with ultrasound tomography: a comparison with non-contrast MRI

**DOI:** 10.1186/bcr3759

**Published:** 2015-11-05

**Authors:** Elizabeth O'Flynn, Jeremie Fromageau, Minty Ledger, Alessandro Messa, Ashley D'Aquino, Minouk Schoemaker, Maria Schmidt, Neb Duric, Anthony Swerdlow, Jeff Bamber

**Affiliations:** 1 Insititute of Cancer Research and Royal Marsden Hospital, Sutton, UK; 2 Karmanos Institute, Detroit, MI, USA

## Introduction

Ultrasound tomography (UST) is an emerging whole breast 3D imaging technique that obtains quantitative tomograms of speed of sound (as well as other properties) of the entire breast. The measured parameter is the volume averaged speed of sound (VASS) [1,2]. It improves on mammography by measuring density at each voxel and holds promise as a cheap, patient-acceptable, non-ionising radiation method to evaluate density. This study was to evaluate the technique of UST and compare VASS with percentage water density from non-contrast MRI.

## Methods

This single-centre cross-sectional trial had research ethics committee approval. Fifty healthy volunteers from the Generations study [3] (median age 40 years, range 30−64 years) underwent bilateral breast UST. Forty-six underwent MRI using a 2-point Dixon technique [4]. VASS and percentage water density measurements were evaluated in both beasts and compared with Pearson's correlation coefficient.

## Results

Mean VASS measurements for the cohort were 1446 ± 148 ms^−1 ^(range 1434−1541 ms). There was high similarity between measurements from the right and left breasts (1463 ± 29 ms^−1^, 1459 ± 29 ms^−1 ^respectively (*p *= 0.516)) (ICC = 0.98). Mean percentage water density for the cohort was 34.6 ± 14.5% (range 13.5−74.4%) with good right-to-left consistency (35.7 ± 15.3%, 34.4 ± 14.6% respectively (*p *= 0.55)). There was excellent correlation between VASS and percentage water density (*r*^2 ^= 0.97, *p* < 0.0001).

## Conclusion

UST holds promise as a robust, reliable and accurate technique to evaluate breast density without the use of ionising radiation and has additional benefits of lower cost and greater patient acceptability.
